# Ultra-Processed Food Consumption and Domain-Specific Quality of Life in Postmenopausal Women: Associations with Mobility and Mental Health

**DOI:** 10.3390/healthcare14060791

**Published:** 2026-03-20

**Authors:** Byung Soo Kwan, Jung-Hwan Cho, Jun Young Kim, Hye In Kim, Nak Gyeong Ko, Ji Eun Park

**Affiliations:** 1 Department of Internal Medicine, Samsung Changwon Hospital, Sungkyunkwan University School of Medicine, Changwon 51353, Republic of Korea; kbs2459@naver.com (B.S.K.); whwjdzl1004@gmail.com (J.-H.C.); jyk2412@gmail.com (J.Y.K.); hyein11.kim@gmail.com (H.I.K.); 2 Department of Research & Support, Samsung Changwon Hospital, Sungkyunkwan University School of Medicine, Changwon 51353, Republic of Korea; 2022230@smc.skku.edu; 3 Department of Obstetrics and Gynecology, Gyeongsang National University Changwon Hospital, Changwon 51472, Republic of Korea; 4 Department of Obstetrics and Gynecology, Gyeongsang National University, Jinju 52727, Republic of Korea; 5 Institute of Medical Science, Gyeongsang National University, Jinju 52727, Republic of Korea

**Keywords:** ultra-processed foods, health-related quality of life, postmenopausal women, mobility, mental health

## Abstract

**Highlights:**

**What are the main findings?**

- Higher ultra-processed food consumption was associated with selective impairments in mobility and anxiety/depression among postmenopausal women, while no consistent association was observed for the overall EQ-5D index score.
- Domain-specific quality-of-life assessments revealed associations that were not captured by summary index measures.

**What are the implications of the main findings?**

- These findings highlight the importance of domain-specific approaches when evaluating diet-related quality-of-life outcomes in postmenopausal women.
- These findings suggest a potential association between higher ultra-processed food intake and poorer mobility and mental health among postmenopausal women.

**Abstract:**

**Background/Objectives:** Ultra-processed food (UPF) consumption is increasing worldwide, yet its domain-specific impact on health-related quality of life (HRQoL) among postmenopausal women remains poorly characterized. This study investigated associations between UPF intake and domain-specific and overall HRQoL in a nationally representative sample of Korean postmenopausal women. **Methods:** Data from the Korea National Health and Nutrition Examination Survey (2013–2021) were analyzed. UPF consumption was assessed using a single 24 h dietary recall and classified according to the NOVA food classification system. HRQoL was evaluated using the five EQ-5D domains and the overall EQ-5D index. Survey-weighted logistic regression models estimated odds ratios (ORs) and 95% confidence intervals (CIs) across UPF intake quartiles, adjusting for socioeconomic and health-related covariates. **Results:** Higher UPF consumption was associated with impairments in specific HRQoL domains rather than a uniform decline across domains. In fully adjusted models, women in the third UPF intake quartile had higher odds of mobility impairment (OR 1.74; 95% CI 1.06–2.86) and anxiety/depression symptoms (OR 1.71; 95% CI 1.06–2.77) than those in the lowest quartile. A significant linear trend was observed for mobility (P-for-trend = 0.012). In contrast, associations with the overall EQ-5D index score were limited and not consistently observed after full adjustment. **Conclusions:** Higher UPF consumption is associated with domain-specific HRQoL impairments, particularly affecting physical mobility and mental health, among postmenopausal women. These findings underscore the importance of domain-specific assessments and suggest that UPF consumption may be related to certain aspects of functional and psychological well-being after menopause.

## 1. Introduction

Ultra-processed food (UPF) consumption has risen globally, becoming a major public health issue [1]. Under the NOVA classification system, which categorizes foods according to the extent and purpose of industrial processing, UPFs are defined as industrial formulations that are high in additives, sugar, and unhealthy fats but low in essential nutrients [2].

Accumulating evidence consistently links high UPF intake to a wide range of adverse health outcomes. Large-scale systematic reviews and meta-analyses have reported that higher consumption of UPFs is associated with increased risks of cardiovascular disease, obesity, type 2 diabetes, metabolic syndrome, and all-cause mortality across diverse populations worldwide [3,4,5,6]. In addition to cardiometabolic conditions, emerging evidence also suggests that UPF-rich dietary patterns may be related to poorer mental health outcomes, including depressive symptoms and psychological distress [5,6]. Despite the expanding literature, most previous studies have primarily focused on clinical endpoints or overall quality-of-life index scores [7], potentially overlooking associations within specific functional and psychological domains. Consequently, evidence on domain-specific aspects of quality of life related to UPF consumption remains limited.

Postmenopausal women represent a population particularly vulnerable to declines in health-related quality of life (HRQoL) due to hormonal changes associated with menopause. The reduction in estrogen levels during this period has been associated with changes in body composition, metabolic health, and musculoskeletal function, which may contribute to limitations in physical mobility and daily activities. In addition, menopause is frequently accompanied by psychological symptoms such as anxiety, depressive mood, and sleep disturbances, which may negatively affect mental well-being. Together, these physiological and psychological changes may increase susceptibility to impairments in both physical and mental domains of HRQoL in this population [8,9]. HRQoL is inherently multidimensional [10], and domain-specific assessments may therefore provide more clinically meaningful insights than a single summary score [11,12]. Evaluating individual domains such as mobility and mental health may be particularly relevant for identifying early functional decline and understanding lifestyle and health-related factors associated with quality of life in postmenopausal women [13,14].

Although a growing number of studies have explored the relationship between ultra-processed food consumption and HRQoL, several aspects of this association remain unclear. In particular, existing research has largely relied on overall quality-of-life index scores, which may provide limited insight into variation across specific functional and psychological domains and are susceptible to ceiling effects in general population samples [15]. In addition, many studies have examined heterogeneous populations without specific consideration of sex or menopausal status [7,16,17], limiting the ability to capture menopause-related vulnerability. Furthermore, evidence from Asian populations remains limited.

Korea provides a relevant context for examining this relationship because dietary patterns in Korea have undergone rapid nutritional transitions in recent decades, with increasing consumption of processed and convenience foods [18]. Therefore, this study investigated the association between UPF intake and domain-specific HRQoL among Korean postmenopausal women using data from the Korea National Health and Nutrition Examination Survey (KNHANES) [18]. Five EQ-5D domains were analyzed: mobility, self-care, usual activities, pain/discomfort, and anxiety/depression [11].

Although the EQ-5D index score was included for comparison, the primary objective was to examine the association between UPF consumption and impairments across individual EQ-5D domains in this population.

## 2. Materials and Methods

### 2.1. Study Design and Data Source

This cross-sectional study used data from the Korean National Health and Nutrition Examination Survey (KNHANES), a nationally representative survey employing a complex, multistage, stratified probability sampling design. For the present analysis, data from multiple survey years were pooled based on the availability of relevant study variables.

All survey procedures were approved by the Institutional Review Board of the Korea Disease Control and Prevention Agency, and written informed consent was obtained from all participants.

### 2.2. Study Population

**Figure 1:**
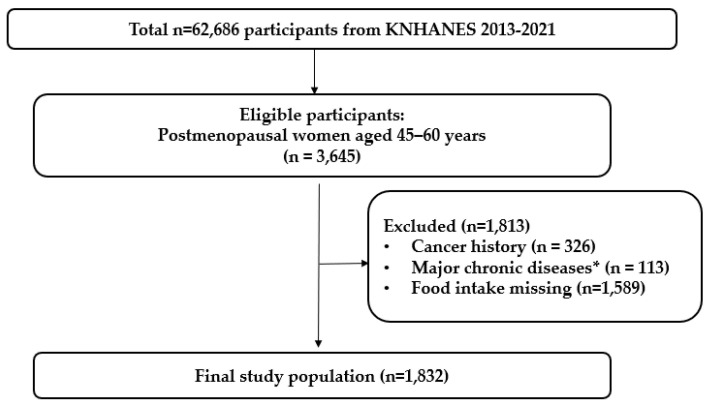
Flow diagram of study participant selection. * Major chronic diseases included myocardial infarction, angina pectoris, stroke, liver cirrhosis, and chronic renal failure.

The study population consisted of postmenopausal women aged 45–60 years identified from the KNHANES between 2013 and 2021. Participants with a history of cancer (n = 326) or major chronic conditions, including myocardial infarction, angina, stroke, liver cirrhosis, and chronic renal failure (n = 113), were excluded. Additionally, participants with missing or zero UPF intake values (n = 1589) or missing covariate data were excluded (Figure 1). The final analytic sample comprised 1832 participants, categorized into quartiles (Q1–Q4) based on UPF consumption.

### 2.3. Assessment of Ultra-Processed Food Consumption

Dietary intake was assessed using a single 24 h dietary recall administered by trained interviewers as part of the KNHANES. All foods and beverages reported during the recall were classified using the NOVA food classification system, which categorizes items into four groups based on the extent and purpose of industrial processing; ultra-processed foods correspond to Group 4 [19].

The NOVA framework was applied with careful consideration of traditional foods and mixed dishes commonly consumed in Korea to ensure appropriate classification within the Korean dietary context [20]. Two trained researchers independently reviewed reported food items and assigned them to NOVA categories, with discrepancies resolved through discussion to reach consensus.

Ultra-processed food intake was quantified for each participant as the proportion of total daily food intake by weight. Specifically, the total weight (g) of ultra-processed food items consumed during the 24 h recall was divided by the total daily food intake (g) and multiplied by 100 to calculate the percentage contribution of ultra-processed foods. Participants were categorized into quartiles based on this percentage, with the lowest quartile used as the reference group in all analyses. This weight-based metric was used because the KNHANES dietary database records food intake primarily in grams.

### 2.4. Assessment of Health-Related Quality of Life

Health-related quality of life was assessed using the EuroQol five-dimension questionnaire (EQ-5D), a standardized, preference-based instrument that evaluates health status across five dimensions and is widely applied in population-based and epidemiological studies [11].

### 2.5. Main Outcomes: EQ-5D Domains

The primary outcomes were impairments across the five EQ-5D domains: mobility, self-care, usual activities, pain/discomfort, and anxiety/depression. Each EQ-5D domain was originally assessed using a three-level response scale (no problems, some problems, and severe problems). For the analysis, responses were dichotomized as no problems (Level 1) versus any problems (Levels 2–3).

EQ-5D domain variables were available across pooled survey years and served as the primary outcomes in this analysis.

### 2.6. Secondary Outcome: EQ-5D Index Score

The EQ-5D index score, calculated using the Korean population–based value set, was analyzed as a secondary outcome to provide complementary information on overall quality of life. Due to data availability, analyses of the EQ-5D index were restricted to the 2019–2020 survey years.

### 2.7. Covariates

Covariates were selected a priori based on availability in KNHANES and established associations with dietary intake and health-related quality of life. Demographic and socioeconomic variables included age (years), educational attainment (≤elementary school, middle school, high school, or ≥college), household income (quartiles from lowest to highest), and marital status (married or not married).

Lifestyle-related factors included smoking status (current smoker or non-smoker), alcohol consumption frequency (less than once per month or at least once per month), and physical activity. Physical activity was defined according to the national physical activity guidelines [21] as aerobic activity only, including ≥150 min per week of moderate-intensity activity, ≥75 min per week of vigorous-intensity activity, or an equivalent combination. Resistance training was not included in this definition.

Body mass index (BMI, kg/m^2^) was calculated from measured height and weight and included as an indicator of adiposity. Total daily energy intake (kcal/day), derived from the 24 h dietary recall, was included to account for overall dietary intake.

The presence of chronic conditions—including hypertension, diabetes mellitus, and dyslipidemia—was determined based on self-reported physician diagnoses from the health interview survey and categorized as yes or no. Duration since menopause (years) was included to account for reproductive and hormonal factors relevant to postmenopausal women.

Covariates were entered sequentially into multivariable regression models to evaluate the robustness of the associations between ultra-processed food consumption and health-related quality of life outcomes.

### 2.8. Statistical Analysis

All statistical analyses used survey procedures to account for the complex, multistage, stratified, and clustered sampling design of the KNHANES. Sampling weights were applied to generate nationally representative estimates for the Korean postmenopausal population.

Baseline characteristics were summarized across quartiles of UPF consumption. Continuous variables were presented as weighted means with standard deviations, whereas categorical variables were presented as frequencies with weighted percentages. Differences across UPF quartiles were evaluated using complex sample analysis of variance for continuous variables and Rao–Scott chi-square tests for categorical variables.

Survey-weighted logistic regression models were employed to evaluate the association between UPF intake quartiles and impairments in each EQ-5D domain, defined as a binary outcome (‘no problems’ vs. ‘any problems’). Odds ratios (ORs) and 95% confidence intervals (CIs) were estimated, using the lowest quartile (Q1) as the reference group. Three hierarchical adjustment models were constructed to assess the stability of the observed associations:

- Model 1 adjusted for age, educational attainment, household income, and marital status.
- Model 2 additionally adjusted for smoking status, alcohol consumption, physical activity, and total energy intake.
- Model 3 additionally adjusted for BMI, hypertension, diabetes, dyslipidemia, and menopausal duration.

This stepwise adjustment strategy was used to evaluate the stability of the associations after sequential control for sociodemographic, lifestyle, and clinical factors. Multicollinearity among covariates was assessed using variance inflation factors (VIFs), and no evidence of problematic multicollinearity was observed (maximum VIF = 3.81).

Linear trends across quartiles were assessed by modeling the mean value of each UPF quartile as a continuous variable. As a secondary analysis, the association between UPF consumption and the EQ-5D index score was analyzed using survey-weighted linear regression with identical covariate adjustments. All statistical tests were two-sided, with a *p*-value of <0.05 indicating statistical significance. All statistical analyses were performed using Stata version 15.1 (StataCorp, College Station, TX, USA).

## 3. Results

### 3.1. Baseline Characteristics of the Study Population

The final analytical sample comprised 1832 postmenopausal women. The median proportion of total food intake from UPFs was 7.99% (interquartile range [IQR]: 3.02–15.85). Table 1 presents the baseline characteristics of participants according to UPF consumption quartiles. Participants in the highest UPF intake quartile (Q4) were significantly younger than those in the lowest quartile (Q1) (54.42 ± 3.58 vs. 54.92 ± 3.38 years; *p* = 0.033). Higher UPF consumption was associated with higher BMI (Q1: 23.38 ± 3.22 vs. Q4: 24.02 ± 3.36; *p* = 0.005) and a higher prevalence of current smoking (*p* = 0.003) and alcohol consumption (*p* < 0.001). No significant differences were observed across quartiles for household income, marital status, or the presence of diabetes and dyslipidemia.

### 3.2. Associations Between Ultra-Processed Food Consumption and Domain-Specific EQ-5D Outcomes

**Figure 2:**
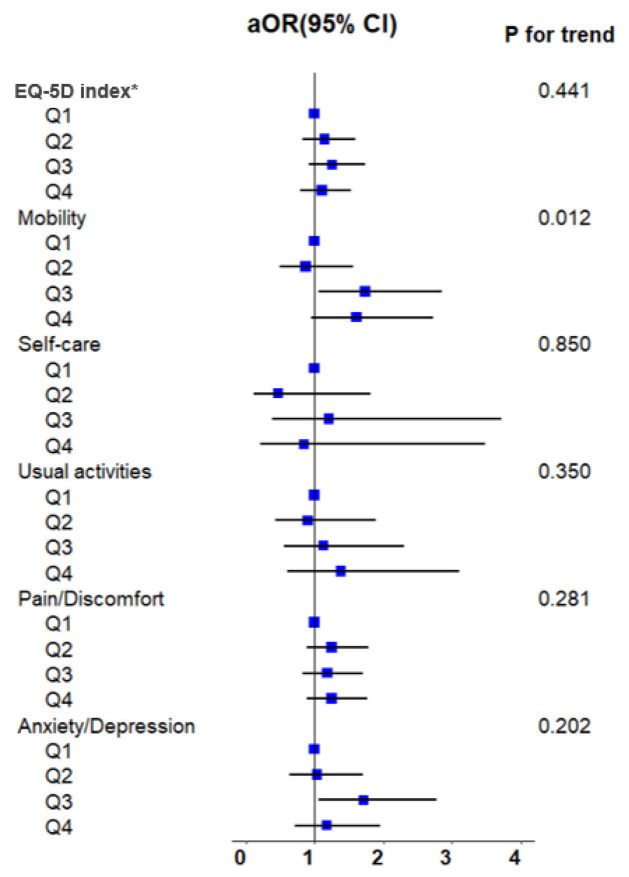
Adjusted odds ratios for low EQ-5D index scores and domain-specific impairments according to quartiles of ultra-processed food consumption among postmenopausal women. Squares represent adjusted odds ratios (ORs), and horizontal lines indicate 95% confidence intervals (CIs). The vertical solid line denotes the null value (OR = 1.0). Odds ratios were estimated using survey-weighted logistic regression models adjusted for age, educational attainment, household income, marital status, smoking status, alcohol consumption, physical activity, total energy intake, body mass index, hypertension, diabetes mellitus, dyslipidemia, and duration since menopause. Q1 represents the reference category. * Due to data availability, analyses of the EQ-5D index were restricted to the 2019–2020 survey years.

Associations between UPF intake and impairments across the five EQ-5D domains are presented in Table 2 and summarized graphically in a forest plot based on the fully adjusted model (Model 3) (Figure 2).

#### 3.2.1. Mobility

Higher UPF consumption was associated with increased odds of mobility impairment. In the fully adjusted model (Model 3), participants in the third UPF consumption quartile (Q3) had significantly higher odds of mobility impairment than those in the lowest quartile (OR 1.74; 95% CI 1.06–2.86). Although the association in the highest quartile (Q4) was of similar magnitude (OR 1.61; 95% CI 0.95–2.73), it did not reach statistical significance. Notably, the test for linear trend across quartiles remained statistically significant (*p* for trend = 0.012).

#### 3.2.2. Anxiety/Depression

A similar pattern was observed in the anxiety/depression domain. In the fully adjusted model, women in Q3 had significantly higher odds of reporting anxiety or depressive symptoms than those in Q1 (OR 1.71; 95% CI 1.06–2.77). The association in Q4 was attenuated (OR 1.18; 95% CI 0.72–1.95) and did not reach statistical significance. The overall linear trend for the anxiety/depression domain was not statistically significant in the fully adjusted model (*p* for trend = 0.202).

#### 3.2.3. Other EQ-5D Domains

No statistically significant associations were observed between UPF consumption and impairments in the self-care, usual activities, or pain/discomfort domains after full adjustment for sociodemographic, lifestyle, and clinical covariates.

### 3.3. Secondary Analysis: EQ-5D Index Score

Results for the EQ-5D index score analyzed as a continuous outcome are presented in Table 3. In Model 1, adjusted for age, educational attainment, household income, and marital status, higher UPF consumption was significantly associated with lower EQ-5D index score (*p* for trend = 0.015). Following full adjustment in Model 3, participants in Q3 maintained a statistically significant, albeit modest, reduction in the EQ-5D index score compared with those in Q1 (β = −0.01; 95% CI −0.03 to −0.002). The association in Q4 was not statistically significant, and the overall linear trend for the index score showed borderline significance (*p* for trend = 0.060).

## 4. Discussion

In this nationally representative study of Korean postmenopausal women, higher UPF consumption was associated with impairments in specific domains of health-related quality of life, particularly mobility and anxiety/depression. Women in the third quartile of UPF intake showed significantly higher odds of reporting mobility impairment (OR, 1.74; 95% CI, 1.06–2.86) and anxiety/depression (OR, 1.71; 95% CI, 1.06–2.77) compared with those in the lowest intake group. No consistent associations were observed for the remaining EQ-5D domains, and associations with the overall EQ-5D index score were limited after full adjustment. Overall, these findings suggest that higher UPF consumption may be related to impairments in selected physical and psychological domains of HRQoL among postmenopausal women.

These findings suggest that mobility represents a particularly sensitive domain reflecting the potential association between UPF consumption and functional health in postmenopausal women. A significant linear trend (*p* for trend = 0.012) was observed, with women in Q3 of UPF intake exhibiting 1.74-fold higher odds of mobility impairment, indicating that physical functioning may be particularly sensitive to diet-related functional changes. During the postmenopausal transition, estrogen deficiency is associated with progressive losses in skeletal muscle mass and strength [22]. Our data suggest that high UPF consumption may be related to greater vulnerability to functional decline during the postmenopausal period.

The mechanisms underlying this association are likely multifactorial. Diets characterized by high UPF intake, which are often nutrient-poor and contain various industrial additives, have been linked to chronic low-grade systemic inflammation [23,24]. Such chronic inflammatory processes can disrupt muscle protein homeostasis and compromise myofibrillar integrity [25]. Furthermore, a high reliance on UPFs may displace essential nutrients, resulting in suboptimal intake of high-quality protein, vitamin D, and dietary fiber, all of which are necessary for maintaining musculoskeletal health. Within the physiological context of menopause, these dietary factors may act as contributing stressors that aggravate age-related declines in physical function. From a public health perspective, these findings suggest that mobility may represent an important domain when examining dietary correlates of quality of life among postmenopausal women.

The findings of this study suggest that anxiety/depression may represent an important domain through which UPF consumption may be associated with health-related quality of life among postmenopausal women. Specifically, women in Q3 of UPF intake exhibited 1.71-fold higher odds of reporting anxiety or depressive symptoms (95% CI, 1.06–2.77) than those in the lowest intake group, indicating that psychological well-being may be particularly sensitive to dietary quality during the postmenopausal transition. This life stage is characterized by a marked decline in estrogen levels, which plays a critical role in neurotransmitter regulation and stress responsiveness, thereby increasing vulnerability to mood disturbances [26].

Several biological mechanisms may underlie this association. Diets high in UPFs are typically characterized by a high glycemic load and low nutritional density, factors linked to increased blood glucose variability and systemic low-grade inflammation, which may contribute to neuroinflammation and dysregulation of neural pathways involved in mood regulation [27,28,29]. In addition, UPF-rich dietary patterns may alter the gut–brain axis through changes in gut microbiota composition, potentially influencing emotional and cognitive function [30]. Within the context of menopause-related neurobiological vulnerability, these dietary factors may act as stressors that aggravate declines in mental well-being. From a public health perspective, these findings highlight mental health as a central component of quality of life in postmenopausal women. Our results suggest that the degree of food processing may represent a modifiable risk factor, offering a potential nonpharmacological approach to alleviating the psychological burden of the menopausal transition and supporting healthier aging trajectories.

The persistence of associations between UPF consumption and impairments in mobility and mental health, even after progressive adjustment for BMI and chronic conditions, represents a noteworthy finding. This finding suggests that the observed relationships are not fully explained by adiposity or pre-existing diseases alone. Rather, these findings indicate that pathways related to dietary quality may contribute to impairments in specific domains of health-related quality of life beyond the effects of excess body weight. Proposed mechanisms include systemic inflammation, oxidative stress, and metabolic dysregulation associated with nutritional imbalance, industrial additives, and the altered food matrix characteristic of ultra-processed products. Although residual confounding cannot be entirely excluded, the persistence of associations in fully adjusted models supports considering UPF consumption as a distinct dietary exposure rather than a proxy for overall energy intake or obesity status.

The observed attenuation of associations in Q4 of UPF consumption relative to Q3 warrants careful interpretation. This non-linear pattern may reflect a complex interplay of underlying factors. First, proactive behavioral modification or reverse causation may explain this pattern, as individuals with emerging subclinical health concerns may have reduced their intake of processed foods, potentially attenuating effect estimates in the highest intake category [31]. Additionally, participants in Q4 tended to be younger (Table 1), a demographic in which functional or psychological impairments may not yet have reached a clinically detectable threshold despite high exposure levels. Although associations did not follow a strictly linear dose–response pattern, the elevated estimates in Q3 may reflect a potential non-linear association across UPF quartiles. This finding implies that UPF consumption may be associated with impairments in specific domains of HRQoL once intake exceeds a certain threshold, rather than increasing in a constant monotonic manner. This interpretation is consistent with the notion that functional and psychological vulnerabilities may first emerge at moderate-to-high exposure levels, whereas cumulative effects in Q4 may be temporarily buffered by younger age or unmeasured lifestyle compensations. Collectively, these findings suggest that the relationship between UPF intake and HRQoL may be complex and warrants further investigation in longitudinal studies.

The pattern of UPF consumption within the Korean dietary context warrants closer examination. Unlike many Western populations, in which UPF intake is largely driven by discretionary snacks, processed meats, and sugar-sweetened beverages, major sources of UPFs in Korea frequently include meal replacements such as instant noodles and ready-to-eat meals [31]. These products, which are typically high in sodium and refined carbohydrates, are often consumed as dietary staples rather than as occasional snacks. Among postmenopausal women, reliance on convenience-based meals may more substantially displace traditional, nutrient-dense Korean dietary patterns—characterized by vegetables, legumes, and fermented foods [32]—during a period of heightened physiological vulnerability. Such dietary shifts may contribute to micronutrient inadequacy and chronic low-grade inflammation, which may partly explain the observed impairments in mobility and mental health. Accordingly, these findings underscore the context-dependent health implications of food processing and highlight the importance of population-specific evidence to inform culturally appropriate dietary recommendations that preserve quality of life in aging populations. In this context, public health efforts such as improving nutrition education and promoting dietary patterns centered on minimally processed foods may help reduce excessive reliance on ultra-processed foods.

This study leverages nationally representative, pooled data from the KNHANES between 2013 and 2021, providing robust statistical power and supporting the generalizability of the findings to Korean postmenopausal women. Focusing exclusively on this demographic, which is characterized by heightened physiological and psychological vulnerability, allowed the analysis to provide population-specific insights that may be diluted in studies of more heterogeneous adult populations.

An important methodological strength of this study is the domain-specific assessment of health-related quality of life. Examining individual EQ-5D dimensions rather than relying solely on a composite index score allowed the study to identify selective impairments in mobility and mental health that could be obscured by summary measures. This approach helps mitigate ceiling effects commonly observed in overall quality-of-life indices in general population samples and enhances sensitivity for detecting early functional decline.

Despite these strengths, some limitations should be acknowledged. The cross-sectional design precludes causal inference, and reverse causality—whereby individuals with poorer quality of life may turn to ultra-processed “comfort foods”—cannot be excluded. Nevertheless, the observed associations are consistent with previous evidence linking dietary quality to musculoskeletal and mental health outcomes.

Furthermore, dietary intake was assessed using a single 24 h recall, which may not adequately reflect long-term habitual intake. Day-to-day variation in food consumption may result in exposure misclassification. In large-scale epidemiological studies, random within-person variation in dietary reporting typically biases associations toward the null, suggesting that the observed estimates may be conservative. Additionally, analyses of the EQ-5D index score were limited to the 2019–2020 survey years due to data availability, resulting in a smaller sample size for this secondary analysis. Finally, another limitation is that each EQ-5D domain is assessed using a single-item question, which may limit the ability to capture the full complexity of constructs such as anxiety or depression compared with multi-item psychological instruments.

## 5. Conclusions

In conclusion, higher UPF consumption among Korean postmenopausal women is associated with impairments in specific dimensions of health-related quality of life—particularly mobility. Associations with the anxiety/depression domain were less consistent and should be interpreted cautiously. These findings highlight the value of examining domain-specific dimensions of HRQoL in addition to overall index measures. Future longitudinal studies are warranted to clarify potential causal pathways and examine the long-term impact of dietary processing on multidimensional quality of life in aging populations.

## Figures and Tables

**Table 1:** Baseline characteristics of postmenopausal women according to quartiles of ultra-processed food intake.

	Quartile of %Total Food Intake from UPF					
	Total (n = 1832)	Q1 (n = 458)	Q2 (n = 458)	Q3 (n = 458)	Q4 (n = 458)	*p*-Value
**%UPF in total food intake, median (IQR)**	7.99 (3.02, 15.85)	1.32 (0.63, 2.06)	5.22 (4.10, 6.37)	10.82 (9.21, 12.96)	25.33 (19.27, 36.80)	
**EQ5D index**	0.95 ± 0.09	0.96 ± 0.07	0.96 ± 0.07	0.94 ± 0.10	0.95 ± 0.09	0.002
**EQ5D domains (any problem), n (%)**						
Mobility	182 (9.63)	38 (7.13)	35 (6.63)	57 (11.60)	52 (13.01)	0.003
Self-care	31 (1.57)	6 (1.44)	7 (0.97)	9 (2.01)	9 (1.85)	0.640
Usual activities	85 (4.59)	18 (4.02)	21 (3.54)	23 (4.75)	23 (6.01)	0.377
Pain/Discomfort	490 (27.16)	111 (23.47)	125 (28.06)	126 (27.63)	128 (29.44)	0.316
Anxiety/Depression	189 (10.34)	39 (8.09)	42 (8.80)	58 (13.22)	50 (11.19)	0.089
**Age (years)**	54.82 ± 3.55	54.92 ± 3.38	55.09 ± 3.62	54.86 ± 3.57	54.42 ± 3.58	0.033
**Educational attainment**						0.032
≤Elementary school	239 (11.44)	51 (9.77)	53 (9.63)	64 (12.23)	71 (14.05)	
Middle school	321 (17.31)	71 (13.86)	87 (20.14)	71 (14.88)	92 (20.34)	
High school	776 (44.95)	205 (48.51)	192 (41.82)	195 (45.51)	184 (43.94)	
≥College	494 (26.30)	131 (27.87)	125 (28.41)	127 (27.39)	111 (21.67)	
**Household income**						0.785
Lowest	153 (8.12)	31 (8.08)	38 (8.36)	39 (8.10)	45 (9.24)	
Lower-middle	426 (22.94)	105 (21.39)	98 (21.48)	106 (23.15)	117 (25.61)	
Upper-middle	516 (27.88)	135 (28.48)	127 (27.74)	123 (27.12)	131 (28.18)	
Highest	731 (41.06)	187 (43.36)	191 (42.42)	189 (41.63)	164 (36.97)	
**Marital status**						0.484
Married	1811 (98.97)	452 (99.00)	456 (99.52)	450 (98.48)	453 (98.90)	
Not married	21 (1.03)	6 (1.00)	2 (0.48)	8 (1.52)	5 (1.10)	
**Smoking status**						0.003
Smokers	79 (4.44)	12 (2.49)	19 (3.70)	4 (3.48)	34 (7.98)	
Non-smokers	1753 (95.56)	446 (97.51)	439 (96.30)	444 (96.52)	424 (92.02)	
**Alcohol consumption**						<0.001
At least once per month	759 (43.51)	157 (34.46)	164 (38.81)	178 (41.19)	260 (59.19)	
Less than once per month	1068 (56.49)	300 (65.54)	293 (61.19)	280 (58.81)	195 (40.81)	
Physical activity *						0.246
High	804 (44.01)	187 (41.83)	203 (44.55)	222 (48.15)	192 (41.55)	
Low	1027 (55.99)	270 (58.17)	255 (55.45)	236 (51.85)	266 (58.45)	
**Total energy intake, kcal/day**	1816.33 ± 704.40	1858.95 ± 724.53	1816.59 ± 686.52	1837.48 ± 739.00	1753.77 ± 660.94	0.083
**Body mass index (kg/m^2^)**	23.70 ± 3.31	23.38 ± 3.22	23.57 ± 3.07	23.84 ± 3.52	24.02 ± 3.36	0.005
**Hypertension**						0.038
Yes	335 (18.54)	93 (22.09)	64 (13.79)	94 (18.83)	84 (19.34)	
No	1497 (81.46)	365 (77.91)	394 (86.21)	364 (81.17)	374 (80.66)	
**Diabetes mellitus**						0.441
Yes	101 (5.63)	32 (7.46)	23 (5.28)	25 (4.99)	21 (4.80)	
No	1731 (94.37)	426 (92.54)	435 (94.72)	433 (95.01)	437 (95.20)	
**Dyslipidemia**						0.169
Yes	434 (23.64)	119 (27.88)	104 (22.03)	109 (23.25)	102 (21.43)	
No	1398 (76.36)	339 (72.12)	354 (77.97)	349 (76.75)	356 (78.57)	
**Duration since menopause (years)**	5.66 ± 4.39	5.65 ± 4.47	5.75 ± 4.29	5.67 ± 4.22	5.59 ± 4.55	0.800

* High physical activity is defined as 150 min/week of moderate-intensity aerobic physical activity, 75 min/week of vigorous-intensity aerobic physical activity, or 150 min/week of a combination of moderate- and vigorous-intensity aerobic activities. Otherwise, physical activity is low. Values are presented as the median (interquartile range) or mean ± standard deviation or number (%) by descriptive or frequency analysis. The *p*-values are calculated using complex sample analysis of variance or Rao–Scott chi-square tests.

**Table 2:** Associations between ultra-processed food consumption and EQ-5D index score and domain-specific health-related quality of life outcomes among postmenopausal women.

	Quartile of %Total Food Intake from UPF				
	Q1	Q2	Q3	Q4	*p* for Trend
Outcome: EQ-5D index (lowest 20%) *					
No. of cases (%)	142 (29.54)	151 (33.29)	160 (35.56)	152 (34.81)	
Crude	reference	1.19 (0.87, 1.63)	1.32 (0.97, 1.79)	1.27 (0.93, 1.73)	0.098
Model 1	reference	1.18 (0.86, 1.62)	1.28 (0.93, 1.75)	1.18 (0.86, 1.62)	0.251
Model 2	reference	1.15 (0.83, 1.58)	1.27 (0.92, 1.74)	1.12 (0.81, 1.54)	0.407
Model 3	reference	1.15 (0.83, 1.60)	1.26 (0.92, 1.73)	1.11 (0.80, 1.53)	0.441
**Outcome: EQ-5D domain (Mobility)**					
No. of cases (%)	38 (7.13)	35 (6.63)	57 (11.60)	52 (13.01)	
Crude	reference	**0.93 (0.54, 1.59)**	**1.71 (1.08, 2.72)**	**1.95 (1.21, 3.13)**	**<0.001**
Model 1	reference	**0.87 (0.50, 1.51)**	**1.66 (1.04, 2.65)**	**1.69 (1.04, 2.77)**	**0.006**
Model 2	reference	**0.82 (0.47, 1.45)**	**1.72 (1.06, 2.77)**	**1.54 (0.92, 2.58)**	**0.016**
Model 3	reference	**0.87 (0.49, 1.56)**	**1.74 (1.06, 2.86)**	**1.61 (0.95, 2.73)**	**0.012**
**Outcome: EQ-5D domain (Self-care)**					
No. of cases (%)	6 (1.44)	7 (0.97)	9 (2.01)	9 (1.85)	
Crude	reference	0.67 (0.20, 2.29)	1.40 (0.45, 4.39)	1.30 (0.39, 4.32)	0.455
Model 1	reference	0.66 (0.19, 2.31)	1.29 (0.42, 4.00)	1.05 (0.31, 3.61)	0.694
Model 2	reference	0.51 (0.13, 2.02)	1.27 (0.40, 3.99)	0.91 (0.21, 3.82)	0.818
Model 3	reference	0.47 (0.12, 1.82)	1.21 (0.39, 3.72)	0.85 (0.21, 3.48)	0.850
**Outcome: EQ-5D domain (Usual activities)**					
No. of cases (%)	18 (4.02)	21 (3.54)	23 (4.75)	23 (6.01)	
Crude	reference	0.88 (0.44, 1.76)	1.19 (0.60, 2.37)	1.53 (0.75, 3.12)	0.177
Model 1	reference	0.89 (0.43, 1.82)	1.12 (0.56, 2.22)	1.36 (0.64, 2.89)	0.341
Model 2	reference	0.88 (0.42, 1.82)	1.10 (0.54, 2.23)	1.32 (0.59, 2.96)	0.403
Model 3	reference	0.90 (0.43, 1.89)	1.13 (0.55, 2.31)	1.38 (0.61, 3.11)	0.350
**Outcome: EQ-5D domain (Pain/Discomfort)**					
No. of cases (%)	111 (23.47)	125 (28.06)	126 (27.63)	128 (29.44)	
Crude	reference	1.27 (0.91, 1.79)	1.24 (0.88, 1.76)	1.36 (0.97, 1.90)	0.098
Model 1	reference	1.27 (0.90, 1.79)	1.20 (0.85, 1.71)	1.28 (0.91, 1.79)	0.225
Model 2	reference	1.25 (0.88, 1.76)	1.19 (0.84, 1.70)	1.25 (0.89, 1.77)	0.262
Model 3	reference	1.25 (0.88, 1.79)	1.19 (0.83, 1.70)	1.25 (0.88, 1.77)	0.281
**Outcome: EQ-5D domain (Anxiety/Depression)**					
No. of cases (%)	39 (8.09)	42 (8.80)	58 (13.22)	50 (11.19)	
Crude	reference	**1.10 (0.67, 1.78)**	**1.73 (1.08, 2.76)**	**1.43 (0.89, 2.30)**	**0.043**
Model 1	reference	1.06 (0.65, 1.74)	1.67 (1.03, 2.70)	1.32 (0.81, 2.15)	0.101
Model 2	reference	1.04 (0.63, 1.71)	1.67 (1.03, 2.72)	1.16 (0.71, 1.91)	0.245
Model 3	reference	1.04 (0.64, 1.70)	1.71 (1.06, 2.77)	1.18 (0.72, 1.95)	0.202

Model 1: Adjusted for age, educational attainment, household income, and marital status. Model 2: Adjusted for age, educational attainment, household income, marital status, smoking status, alcohol consumption, physical activity, and total energy intake. Model 3: Adjusted for age, educational attainment, household income, marital status, smoking status, alcohol consumption, physical activity, total energy intake, body mass index, hypertension, diabetes mellitus, dyslipidemia, and duration since menopause. * Lowest 20% indicates participants in the lowest quintile of the EQ-5D index score. Odds ratios (ORs) and 95% confidence intervals (CIs) were estimated using survey-weighted logistic regression models. The *p* for trend is calculated using survey-weighted logistic regression analysis. Bold values indicate statistical significance (*p* < 0.05).

**Table 3:** Associations between ultra-processed food consumption and EQ-5D index score according to quartiles of intake among postmenopausal women.

	Quartile of %Total Food Intake from UPF				
	Q1	Q2	Q3	Q4	*p* for Trend
Crude	reference	**−0.004 (−0.01, 0.01)**	**−0.02 (−0.03, −0.004)**	**−0.02 (−0.03, −0.004)**	**0.002**
Model 1	reference	**−0.002 (−0.01, 0.01)**	**−0.02 (−0.03, −0.003)**	**−0.01 (−0.02, 0.001)**	**0.015**
Model 2	reference	−0.001 (−0.01, 0.01)	−0.01 (−0.03, −0.002)	−0.01 (−0.02, 0.004)	0.053
Model 3	reference	−0.001 (−0.01, 0.01)	−0.01 (−0.03, −0.002)	−0.01 (−0.02, 0.005)	0.060

Model 1: Adjusted for age, educational attainment, household income, and marital status. Model 2: Adjusted for age, educational attainment, household income, marital status, smoking status, alcohol consumption, physical activity, and total energy intake. Model 3: Adjusted for age, educational attainment, household income, marital status, smoking status, alcohol consumption, physical activity, total energy intake, body mass index, hypertension, diabetes mellitus, dyslipidemia, and duration since menopause. The *p* for trend is calculated using survey-weighted linear regression. Bold values indicate statistical significance (*p* < 0.05).

## Data Availability

The data used in this study were derived from publicly available data from the Korean National Health and Nutrition Examination Survey (KNHANES), conducted by the Korea Disease Control and Prevention Agency (https://knhanes.kdca.go.kr/ (accessed on 1 August 2025)).

## Abbreviations

The following abbreviations are used in this manuscript:

BMI	Body Mass Index
CI	Confidence Interval
EQ-5D	EuroQol Five-Dimension Questionnaire
HRQoL	Health-Related Quality of Life
IQR	Interquartile Range
KNHANES	Korea National Health and Nutrition Examination Survey
NOVA	NOVA Food Classification System
OR	Odds Ratio
Q1–Q4	Quartiles 1 to 4
UPF	Ultra-Processed Food(s)
